# Translation of Epigenetics in Cell-Free DNA Liquid Biopsy Technology and Precision Oncology

**DOI:** 10.3390/cimb46070390

**Published:** 2024-06-27

**Authors:** Wan Ying Tan, Snigdha Nagabhyrava, Olivia Ang-Olson, Paromita Das, Luisa Ladel, Bethsebie Sailo, Linda He, Anup Sharma, Nita Ahuja

**Affiliations:** 1 Department of Surgery, Yale School of Medicine, New Haven, CT 06520-8000, USA; wanying.tan@yale.edu (W.Y.T.); paromita.das@yale.edu (P.D.); luisa.ladel@nuvancehealth.org (L.L.); bethsebie.sailo@yale.edu (B.S.); linda.he@yale.edu (L.H.); 2 Department of Internal Medicine, Norwalk Hospital, Norwalk, CT 06850, USA; 3 Hematology & Oncology, Neag Comprehensive Cancer Center, UConn Health, Farmington, CT 06030, USA; 4 Department of Pathology, Yale School of Medicine, New Haven, CT 06520-8000, USA; 5 Biological and Biomedical Sciences Program (BBS), Yale University, New Haven, CT 06520-8084, USA

**Keywords:** epigenetics, epigenomics, cell-free DNA, circulating tumor DNA, liquid biopsy, translational medicine, precision oncology, personalized medicine, fragmentomics, cancer disparity, DNA methylation, histone modification, chromatin remodeling, nucleosome positioning, epigenetic drugs, targeted therapy, early cancer detection, cancer screening

## Abstract

Technological advancements in cell-free DNA (cfDNA) liquid biopsy have triggered exponential growth in numerous clinical applications. While cfDNA-based liquid biopsy has made significant strides in personalizing cancer treatment, the exploration and translation of epigenetics in liquid biopsy to clinical practice is still nascent. This comprehensive review seeks to provide a broad yet in-depth narrative of the present status of epigenetics in cfDNA liquid biopsy and its associated challenges. It highlights the potential of epigenetics in cfDNA liquid biopsy technologies with the hopes of enhancing its clinical translation. The momentum of cfDNA liquid biopsy technologies in recent years has propelled epigenetics to the forefront of molecular biology. We have only begun to reveal the true potential of epigenetics in both our understanding of disease and leveraging epigenetics in the diagnostic and therapeutic domains. Recent clinical applications of epigenetics-based cfDNA liquid biopsy revolve around DNA methylation in screening and early cancer detection, leading to the development of multi-cancer early detection tests and the capability to pinpoint tissues of origin. The clinical application of epigenetics in cfDNA liquid biopsy in minimal residual disease, monitoring, and surveillance are at their initial stages. A notable advancement in fragmentation patterns analysis has created a new avenue for epigenetic biomarkers. However, the widespread application of cfDNA liquid biopsy has many challenges, including biomarker sensitivity, specificity, logistics including infrastructure and personnel, data processing, handling, results interpretation, accessibility, and cost effectiveness. Exploring and translating epigenetics in cfDNA liquid biopsy technology can transform our understanding and perception of cancer prevention and management. cfDNA liquid biopsy has great potential in precision oncology to revolutionize conventional ways of early cancer detection, monitoring residual disease, treatment response, surveillance, and drug development. Adapting the implementation of liquid biopsy workflow to the local policy worldwide and developing point-of-care testing holds great potential to overcome global cancer disparity and improve cancer outcomes.

## 1. Introduction

Liquid biopsy has emerged as a transformative minimally invasive technology in cancer diagnostics, initially devised to detect circulating tumor cells (CTCs) and disseminated tumor cells (DTCs) from peripheral blood and bone marrow, respectively [1]. Liquid biopsy’s evolution from a niche technique to a cornerstone of modern oncology is based on the practicality and adaptability of this technology. In recent years, liquid biopsy has witnessed an explosive surge, extending its reach far beyond blood to encompass a spectrum of bodily fluids such as urine, ascitic fluid, pleural fluid, cerebrospinal fluid, sputum, saliva, and stool. This broadening landscape reflects its capacity to probe an array of circulating biomarkers, both tumor derived and non-tumor derived, including circulating tumor cells (CTC), circulating tumor DNA (ctDNA), circulating tumor RNA, extracellular vesicles such as exosomes [2] and mitochondria [3], cell-free DNA (cfDNA), and circulating cell-free microbial DNA [4]. Importantly, biofluids’ output may change in response to various biological stimuli and environmental factors, empowering liquid biopsy to detect subtle changes indicative of early disease, disease progression, and response to treatment.

The clinical application of cfDNA-based liquid biopsy, mainly using blood samples, is widely accepted for genetic mutation screening in oncology. With the advancement of genomic technologies, common genetic mutations prevalent across various cancer types have been well characterized and cataloged in TCGA and cBioPortal databases. Harnessing molecular data from well-established and characterized databases for developing blood-based assays to identify mutation signatures within cfDNA offers a predictive lens into clinical outcomes and guides targeted therapeutic interventions. Given the inherent cancer heterogeneity, a new era of personalized medicine is needed to improve clinical outcomes. Further identification of unique patterns of changes in the components of biofluids can be harnessed as biomarkers for other clinical applications such as early detection, therapeutic targets, monitoring, and surveillance. Beyond the domain of genetic mutations, epigenetics has emerged as an important player in developing cancer biomarkers. Epigenetics is often referred to as reversible genomic mechanisms that influence gene expressions without altering the sequences of DNA [5]. The dynamic nature of epigenetic modifications gives cells the agility to adapt to environmental changes, and as an early event, they contribute to cellular plasticity and resilience. The versatility of epigenetic modifications makes them a lucrative target for biomarker development in liquid biopsy and paves the way to precision oncology.

The translation of epigenetics in cfDNA liquid biopsy technology has yet to reach the same level of clinical adoption as its genetic counterpart, which is widely translated into clinical practice. This comprehensive review article aims to shed light on the current landscape of epigenetics within cfDNA-based liquid biopsy approaches and its associated challenges to bolster clinical applications in oncology.

## 2. The Role of Epigenetics in Cancer: Beyond Genetic Mutations

The intricate interplay between genetics and epigenetics in cancer biology underscores a profound truth: the cancer story is not written solely in the language of DNA mutations. While genetic mutations have long been spotlighted as the primary culprits in the onset and progression of cancer, this perspective is increasingly recognized as insufficient to comprehend the complex narrative of cancer development fully. Epigenetics, the study of heritable changes in gene expression that do not involve alterations to the underlying DNA sequence, offers a critical lens through which to view the multifaceted nature of cancer [6,7,8,9,10]. It highlights how changes in DNA methylation patterns, histone modifications, and chromatin remodeling play pivotal roles in the emergence and evolution of cancer determinants that can help control the disease. This realization ushers in a paradigm shift, suggesting that the interplay between genetic mutations and epigenetic alterations forms the bedrock upon which the malignant transformation of cells is built, challenging us to broaden our understanding beyond the genetic mutations to encompass the vast epigenetic landscape that shapes cancer’s progression. Since genetics and epigenetics mechanisms work together to govern gene expression, aberrancy in both genetic and epigenetic mechanisms can lead to cellular dysregulation and tissue pathologies. Accumulation of acquired derangements through a multi-step process, accomplished through a combination of genetic mutations as well as epigenetic changes and reprogramming, ultimately leads to the development of the biological hallmarks of cancer, including sustaining proliferative signaling, evading growth suppressors, resisting cell death, enabling replicative immortality, inducing angiogenesis, activating invasion and metastasis, reprogramming energy metabolism, and evading immune destruction, as proposed by Hanahan and Weinberg [11,12]. As this process continues, neighboring non-cancerous cells lose their capacity to counterbalance the actions of malignant cells, leading to cellular damage, multi-organ failure, and an inability to support life. The inadequacy of genetic mutations to account for cancer’s complexity arises from several key factors that underscore the importance of looking beyond DNA alterations alone [13,14].

### 2.1. Precision Oncology Models in Cancer Development

The quest to understand tumor origins and development remains elusive despite various models, theories, and experimentations [15]. Most current cancer evolution models do not incorporate epigenetic effects of aging, diversity of lifestyle, environmental exposures, and ethnicity as background drivers of mutations. Several lifestyle exposures have been shown to contribute to tumorigenesis, such as alcohol [16], tobacco smoke [17], UV radiation [18], and asbestos [19]. Environmental long-term effects of major catastrophic events, such as the exposure to radiation emitted from atomic bombs and the Chernobyl nuclear plant, were also observed in cancer development [20,21]. Food can also contain carcinogens and be divided into four major groups. The first group is natural products which contain unavoidable carcinogens, such as salted fish. The second group is natural products containing avoidable carcinogens, such as grain contamination with the carcinogenic fungal metabolite aflatoxin, can be circumvented with proper storage. The third group refers to anthropogenic chemicals, such as 2,3,7,8-tetrachlorodibenzo-p-dioxin, that are inadvertently produced during manufacturing and contaminate foodstuffs. The fourth group comprises anthropogenic chemicals intentionally added to foods, such as saccharin or food coloring [22]. The impact of these lifetime exposures may be captured at the epigenetic level and can be inherited by subsequent generations [23]. This phenomenon of epigenetic memory may contribute to the trans-generational inheritance of gene expression in response to experiences in the previous generations [24].

Geospatial disease prevention and control approaches have recently gained increasing attention [25,26]. The impact of geography and the spatial environment, such as living, working, and social conditions, has been recognized as an important factor in disease development [27,28,29,30]. Recognition and detection of location-based patterns and disease trends can help detect unique risk factors specific to the local environment. In cancer development, it has been shown that cancer incidence, survival, and disparities vary depending on the geospatial environment. Environmental epigenetics or social epigenetics are promising avenues yet to be explored in depth. This emerging field may improve precision oncology models [31].

**Figure 1:**
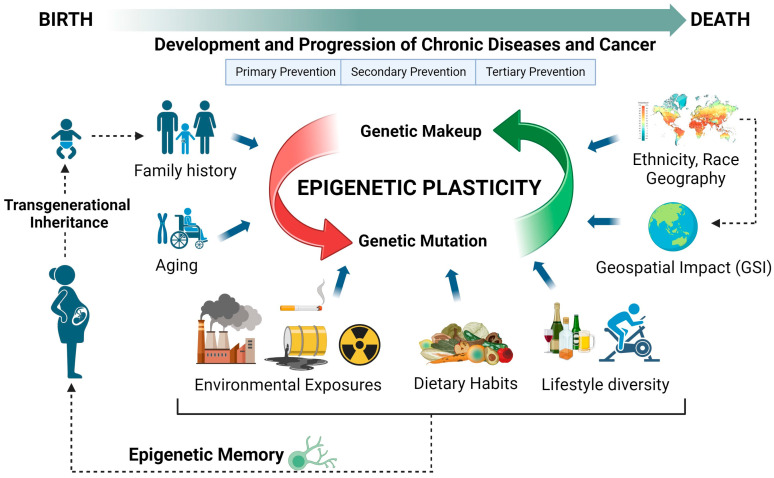
Impact of lifetime exposure on epigenetics plasticity and the development and progression of chronic diseases such as cancer. An individual’s genetic makeup is influenced by ethnicity, race, geography, and family history via trans-generation inheritance. Genes may undergo mutations because of prolonged exposures to the local environment and lifestyle habits, which accumulate throughout a lifetime. These genetic mutations may be passed down through generations. Epigenetic changes are highly dynamic and plastic, influencing gene expressions in response to changes and insults from the local environment. Crucial epigenetic alterations are passed on from ancestors and inherited by subsequent generations. This may lead to the priming of accelerated aging and the development of various diseases.

The challenge to considering these factors in cancer development is the amount of time these factors take to show an impact on tumorigenesis and the need for longitudinal cohorts. The early epigenetic changes and non-invasive approach of liquid biopsy, in parallel to the advances in machine learning and bioanalytical approaches, may provide insights into the impact of these drivers in tumorigenesis. Here, we introduce a model for cancer and chronic disease development rooted in epigenetics, built upon lifetime exposures, and encompassing all mutation drivers, aiming to offer valuable insights for precision medicine depicted in Figure 1.

Aging is another important factor in cancer development that is usually overlooked. The relationship between aging and an increased risk of developing cancer is well established [32]. Cancer primarily affects older individuals, yet research using aged animal models is infrequent, and older adults are often inadequately included in cancer clinical trials [33,34,35]. Aging significantly impacts cancer development through various biological mechanisms and processes, such as accumulation of genetic mutations over time, telomere shortening, reduced effectiveness of DNA repair mechanisms, changes in hormone levels, immunosenescence, chronic inflammation, stem cell exhaustion, and epigenetic alterations [36]. Tissue microenvironmental changes also occur during aging, affecting the extracellular matrix and intercellular communication and influencing cancer initiation and progression. In mammals, aging is associated with widespread and targeted changes in DNA methylation patterns across the genome [34,35,37,38]. Although there is a global loss in methylation during aging, CpG island methylation increases and progresses to hypermethylation in cancer. Promoter hypermethylation has been linked to the inactivation of tumor-suppressor genes [38]. Age-related aberrancy in methylation is one of the earliest changes that marks the increased risk of cancer development [34]. For example, age-dependent increases in methylation of several genes, including ER [32], IGF2 [33], N33 [34], and MyoD [35], were shown to progress to hypermethylation in colorectal cancer tumorigenesis [35,39].

Furthermore, aging is characterized by a widespread reduction in histone proteins and extensive alterations in chromatin structure across different aging models [40]. The aging process and lifetime accruing of cellular injury and epigenetic modifications in cancer development may potentially provide a window of opportunity to detect early changes in cellular behavior, which may possess signatures that reflect susceptibility to tumorigenesis. Liquid biopsy technology may change the current primary and secondary cancer prevention approach, including disease monitoring and surveillance.

### 2.2. Tumors Heterogeneity: Driver and Passenger Mutations

Traditionally, tumor heterogeneity is broadly divided into intertumoral and intratumoral heterogeneity. Intertumoral heterogeneity refers to diversity between patients harboring tumors of the same histological type due to patient-specific factors such as germline mutations, somatic mutations, and environmental exposures. Intratumoral heterogeneity refers to diversity within tumors, which can occur temporally and spatially. Over time, cancer cells can acquire more mutations, which may lead to dynamic changes in the tumor molecular profile and the propagation of its malignant potential. Tumor subpopulations with distinct features may arise within the same tumor and across different locations in the body when dispersed to the metastatic sites [41]. In the study of tumor heterogeneity, the genetic basis of cancer has been the primary focus in understanding the evolution and progression of the disease [42].

Based on the traditional tumor heterogeneity concept, current cancer evolution models rely heavily on genomic perturbations through the concept of driver and passenger mutations, which overlooks the impact of other drivers of cancer evolution [13]. This has led to increased clinical trials based on genetic mutations in recent years. However, the percentage of developmental drugs that reach clinical significance has been slow, given concerns about safety or lack of efficacy signals in trials [43,44]. One of the major reasons for the toxicity and lack of efficacy of developmental anticancer drugs is target mutation mischaracterization or targeting mutations that are not essential for cancer cell survival [45]. Additionally, compared to its genetic counterpart, the significance of epigenetics as a driver mutation in tumor heterogeneity may be another crucial factor contributing to the lack of consistency in treatment response [13,46]. Numerous studies have shown a high frequency of mutations in genes encoding proteins that regulate epigenomic processes in cancers, and it is well established that aberrations of epigenetics machinery play a key role in tumorigenesis [42,47,48]. However, the significance of many of these genetic changes on the cancer epigenome, whether as drivers or passengers, is still being explored [48]. Understanding and targeting epigenetic heterogeneity could improve clinical outcomes in cancer treatment [49].

### 2.3. Epigenetic Regulation of Cellular Plasticity

Cell plasticity refers to the ability of a cell to change its fate in response to various intrinsic or extrinsic factors. Both normal and cancer cells possess the ability to acquire new cellular phenotypes and demonstrate cellular plasticity. Increased plasticity is crucial for physiological tissue regeneration and repair seen in normal tissue and adult stem cell activity and cancer development [50]. The potency of cellular plasticity is also dictated by cellular senescence which is a hallmark of biological and chronological aging [51,52]. Cellular senescence plays a key role in the regenerative capacity of tissues and may serve as a potential indicator of pathological tissue states and age-related diseases [51,52,53]. In the context of cancer, cells are thought to have higher plasticity than terminally differentiated normal cells, driven by various epigenetic states to switch between different cellular phenotypes to adapt to environmental challenges [50,54]. Epigenetics has emerged as a key factor in cellular plasticity. The interplay of epigenetic states and gene regulation is central to understanding cellular plasticity. However, their specific relationship is still unclear [55]. Epigenetics factors such as DNA methylation [56], histone modification [57], and chromatin remodeling [58] play an important role in the regulation of epithelial–mesenchymal transition (EMT) and are discussed in depth in other articles [59,60].

Cancer plasticity is influenced by dynamic epigenetic changes, which can lead to drug resistance and poor outcomes. Genetic and epigenetic mutations occur in cancer cells during therapy, allowing them to survive harsh conditions during metastasis and disease progression [61]. One approach to treating cancer is targeting the plasticity of the tumor microenvironment (TME). However, this is a complex task due to the multifaceted nature of the stroma. For instance, pancreatic cancer is particularly deadly due to its resistance to conventional chemotherapeutic regimens, which can be attributed to the highly desmoplastic stroma in its TME [62]. Additionally, the highly dynamic nature of the epithelial-to-mesenchymal transition (EMT) contributes to the aggressiveness and chemoresistance of pancreatic cancer [63]. Furthermore, cancer survivors may experience ‘accelerated aging’ after treatment, which alters the degree of plasticity for tissue regeneration and repair [64]. Capturing epigenetic alterations in addition to genetic mutations through non-invasive liquid biopsy approaches can detect drug resistance early and predict therapeutic efficacy. Understanding the origins of these changes may help explain the variations and unpredictable clinical outcomes in treatment responses.

### 2.4. Role of Epigenetics in Tissue-of-Origins (TOO)

Tissue biopsy remains the gold standard due to its reliability and accuracy in disease diagnosis and surveillance despite the emerging popularity of liquid biopsy as a promising test. Identifying the tissue of origin (cancer site of origin) remains an unaddressed barrier in the widespread use of liquid biopsy for population screening and risk stratification in populations. As an example, the ability to distinguish TOO is crucial in high-risk individuals with familial risk or an underlying genetic susceptibility at risk for multiple cancers, especially since this group would benefit most from liquid biopsy for screening and monitoring at a young age. For example, KRAS mutations may be detected in several types of cancers, such as small-cell lung, colorectal, and pancreatic cancer [65]. In addition to identifying which diseased organs the cfDNA originated from, it is also important for markers to distinguish normal from diseased tissues.

Epigenetics are emerging biomarkers of TOO. DNA methylation is the epigenetic feature that has been studied the most and is the most promising as an epigenetic TOO biomarker [66,67,68]. It has been found that different cell types have distinct DNA methylation patterns to retain cell identity [69,70]. Tissue-specific DNA methylation markers are sensitive for the blood-based detection of tissue cell death [71,72]. This will help delineate cancer-specific origins, thereby increasing diagnostic accuracy.

## 3. Fundamentals of Epigenetics in cfDNA-Based Liquid Biopsy

As part of the natural history of the disease, cancer cells are generally thought to have a high turnover rate and may circulate freely in blood. CfDNA is believed to be mainly released from cells via apoptosis, necrosis, and possibly from active secretion [73]. Based on these concepts, the alluring idea of detecting cfDNA released from cancer cells became one of the core principles of liquid biopsy. Specifically, cfDNA refers to non-encapsulated DNA that circulates freely in body fluids such as plasma, cerebrospinal fluid, pleural fluid, urine, and saliva [74]. cfDNA was first discovered by Mandel and Métais in 1948 [75]. Stroun et al., 1987 then showed the presence of cfDNA in cancer patients [76]. The fraction of cfDNA derived from tumor cells is named circulating tumor DNA (ctDNA). The more advanced cancers are expected to have more circulating cancer cells, ctDNA, and cfDNA that can be detected in blood [77].

**Figure 2:**
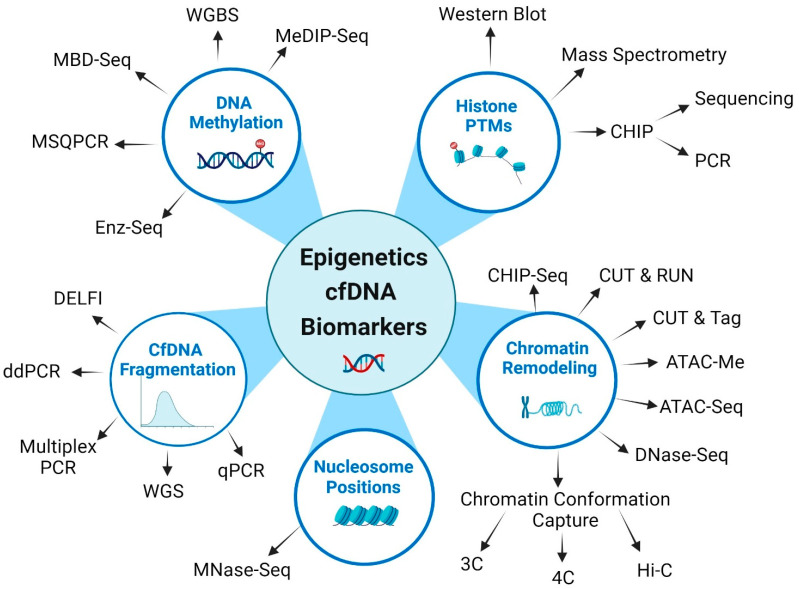
Epigenetic mechanisms and cfDNA biomarkers with detection methods in liquid biopsy. Arrows show the detection method. Abbreviations: Seq: sequencing, MBD-Seq: methyl-CpG-binding domain sequencing, MSQPCR: methylation-specific polymerase chain reaction, WGBS: whole genome bisulfite sequencing, Enz-Seq: enzymatic converted sequencing, MeDIP-Seq: methylated DNA immunoprecipitation followed by sequencing, CHIP: chromatin immunoprecipitation, MNase-Seq: micrococcal nuclease digestion with deep sequencing, CUT&RUN: cleavage under targets and release using nuclease, CUT&Tag: cleavage under targets and tagmentation, ATAC-Seq: assay for transposase-accessible chromatin with high-throughput sequencing, DNase-Seq: DNase I hypersensitive sites sequencing, qPCR: quantitative polymerase chain reaction, ddPCR: droplet digital polymerase chain reaction, DELFI: DNA evaluation of fragments for early interception.

The cfDNA-based epigenetic biomarkers can be categorized conceptually as related to epigenetic regulation or mechanisms. Biomarkers related to epigenetic regulation that can be captured through liquid biopsy are genes, proteins, and enzymes in the epigenetic signaling pathways that modulate the control of epigenetic alterations. These molecules execute epigenetic changes of writing, reading, and erasing processes, such as DNMT, HIDAC, EZH2, and SWF complex genes or proteins [78]. Another aspect of epigenomics is identifying the apparatus and mechanisms that work together to execute epigenetic regulation. This machinery is known as epigenetic mechanisms. Several epigenetic mechanisms have been identified, such as DNA methylation, histone modifications, nucleosome positioning, cfDNA fragmentation, and chromatin structural changes, as depicted in Figure 2**.** These epigenetic mechanisms which can be detected in liquid biopsy are described in the following subsections.

For our review, we will be focusing mainly on epigenetic mechanisms. Mutations in epigenetic regulator genes that govern epigenetic mechanisms fall under the category of genomic mutations and will only be briefly discussed in the subsection on epigenetic regulator mutations. Although we conceptualize mutations of epigenetic regulation and epigenetic mechanisms as two separate categories, they are not strictly exclusive. Epigenetic regulation and downstream mechanistic changes are intricately intertwined. For instance, the open form of chromatin (an epigenetic mechanism) allows for the transcription of genes that may serve as ‘writers’, ‘readers’, or ‘erasers’ (epigenetic regulators), resulting in the regulation of chromatin remodeling (epigenetic mechanism). Analytical methods of each epigenetic feature will be discussed in a separate section below.

### 3.1. Epigenetic Regulator Mutations

Mutation of epigenetic regulator genes can be detected through cfDNA in liquid biopsy using PCR-based tests or sequencing methods in genomic studies. These genes encode proteins and enzymes described as ‘writers’, ‘readers’, and ‘erasers’ involved in signaling pathways mediating epigenetic changes in the cell [79]. For example, DNA methyltransferases (DNMTs), histone methyltransferases (HMTs), histone acetyltransferases (HATs), histone lysine demethylases (KDMs), and histone deacetylases (HDACs) are common enzymes regulating methylation and acetylation of DNA and histones. Detailed descriptions of the roles of these genes are discussed in greater depth in other review articles [80]. Mutations of epigenetic regulator genes have been reported in many cancers, such as gastric cancer [81], acute myeloid leukemia (AML) [82], lymphomas [83], and chronic myelomonocytic leukemia (CMML) [84]. Epigenetic regulator mutations are also postulated to play a role in appendiceal cancers [78]. Among the various epigenetic regulators, one of the most common mutations occurs in DNMT3A. For example, approximately 50% of the DNMT3A mutations occur in the catalytic domain at position R882 (most commonly R882H) [85,86]. DNMT3A mutations are present in up to 20% of AML cases associated with poor prognosis [87]. The dysregulation of H3K27 trimethylation (H3K27me3) due to mutations in EZH2, a histone methyltransferase, is emerging as an important driver in human tumorigenesis and has been reported in lymphomas and melanoma [88,89].

### 3.2. Epigenetic Mechanisms in Cancers

#### 3.2.1. DNA Methylation

DNA methylation is an important heritable epigenetic modification that plays a massive role in the regulation of gene expression and is frequently known as the ‘repressor’ or ‘silencer’ of gene expression. The process of DNA methylation occurs when the methyl group is transferred from S-Adenosyl Methionine (SAM) to 5′ carbon of cytosine nucleotides adjacent to guanines (CpG dinucleotides) forming 5-methylcytosine (5-mC) via the DNMT enzymes [90]. Methylation occurs across the genome at transcriptional start sites (TSS) with or without CpG islands (CGI), in gene bodies, at regulatory elements, and at repeat sequences [91]. In normal cells, most CpG dinucleotides are methylated, except CpG islands, which are hypomethylated. CpG islands (CGIs) refer to regions of the genome that contain many CpG dinucleotide repeats, which usually extend from 300 to 3000 base pairs in mammalian genomes [92]. Approximately 70% of vertebrate gene promoters are associated with CGIs [93,94]. They are found in the promoters of all housekeeping genes, and 40% of genes show tissue-specific expression [95]. CGIs have been estimated to constitute 1%–2% of the mammalian genome [96]. Derangement in methylation on CGIs plays a huge role in carcinogenesis.

Aberrations in DNA methylation are characteristics of cancer cells. In cancer cells, genome-wide hypomethylation and hypermethylation of CpG islands are usually seen. Functionally, hypermethylation is associated with silencing tumor suppressor genes (TSG) and developmental regulators, leading to aberrant gene expressions [97]. DNA methylation in promoters of TSG was initially shown in the retinoblastoma tumor suppressor gene (RB1) in patients with retinoblastoma [98]. Subsequently, many other TSG promoters were found to be hypermethylated in many cancers, including colorectal cancers [39,99,100], lung cancers [101], breast cancer [102], pancreatic cancer [103,104], and prostate cancer [105]. DNA hypermethylation changes are seen early in carcinogenesis and are tissue specific, allowing for identifying tissue of origin [106,107]. These characteristics make DNA methylation a promising biomarker application, especially early detection in liquid biopsy. Multiple non-invasive tests have been developed based on DNA methylation markers, such as Epi proColon (stool-based test) and Cologuard (Stool-based test) for colorectal cancer [108]. The exciting blood-based multi-cancer early detection (MCED) test by GRAIL Galleri also utilizes cfDNA methylation-based assay [109].

#### 3.2.2. Histone Post-Translational Modifications (PTMs)

Histones are proteins found in the nuclei of eukaryotic cells that form an octamer known as core histones, which serve as a skeleton for DNA binding to support chromosomal structures. Core histones (H3, H4, H2A, and H2B) are wrapped around by 147 DNA base pairs, forming functional units known as nucleosomes, which are then packed together as chromatin [110]. N-terminal amino acid tails protrude from nucleosomes. Both N-terminals and core region histones can undergo post-translational modifications (PTMs) such as phosphorylation, acetylation, ubiquitylation, and methylation [111]. A review of specific histone PTMs is discussed elsewhere [112,113,114]. The balance and stability of histone PTM are crucial in controlling chromatin compaction or signaling to other protein complexes, nucleosome dynamics, and transcriptions to regulate gene expression. The most studied histone PTM as a potential biomarker is methylation. Histone methylation can occur on all basic amino acid residues, such as arginine, lysines, and histidines of histone proteins. Functionally, histone methylation plays a key role as an activator or repressor mark in gene expression and is highly dependent on the core histone and position residues methylated, which also vary in a tissue-specific manner.

Dysregulation of histone modifications and its associated enzymes are often seen in cancers. For example, methylation of the histone H3 at various sites plays different roles in male urogenital cancers [115]. Histone methylation activator marks include H3K4me3 and H3K36me3, whereas repressor marks are H3K27me3 and H3K9me3 [115]. Identification and profiling of histone PTMs and proteins regulating PTMs are important in understanding diseases and in biodiscovery efforts to determine biomarkers screening, treatment, and surveillance. The detection of histone PTM marks can be performed through two major approaches: antibody-based precipitation or mass spectrometry-based proteomics, which can be applied to any tissue type, such as blood [116]. However, most of the current strategies for the clinical translation of histone PTM applications are not based on PTM marks per se. Instead, it is mainly done by detecting mutations in regulatory genes that perform histone methylation and demethylation, which are reviewed in depth elsewhere [117]. With the development of further analytical techniques, identifying specific histone PTM biomarkers holds promising potential in cfDNA-based liquid biopsy for clinical diagnostics, prognostics, and therapeutic targets.

#### 3.2.3. Nucleosome Repositioning and cfDNA Fragmentation

A nucleosome is a globular-shaped structure consisting of core histone proteins wrapped around DNA. Since DNA is a highly negatively charged molecule that readily aggregates, DNA packaging in the form of nucleosomes allows for controlled and regulated DNA compaction in the eukaryotic genome [118,119]. Nucleosomes are arranged in ‘strings-of-beads’ and are interconnected via linker DNA to modulate the state of chromosome structures [119]. Nucleosome positioning refers to the relative position of DNA structure to the histone octamers in the chromatin. The dynamic and constant repositioning of nucleosomes is part of the chromatin remodeling machinery and has emerged as key to understanding the regulation of gene expression. Functionally, nucleosome positioning modulates higher-order chromatin organization and architecture to control the accessibility of protein binding sites crucial for DNA transcription and replication [120]. Genome-wide studies in various organisms showed that promoter regions are depleted in nucleosomes relative to transcribed regions [121,122]. In multicellular organisms, nucleosome positioning showed more variability among cell types and relatively homogenous cell populations such as T cells [123].

One of the most exciting developments in line with the study of nucleosome repositioning is the analysis of fragmentation patterns, known as fragmentomics [124]. The beginning of the era of fragmentomics is driven by the need to better analyze and characterize cfDNA in disease states. It was also suggested that some cfDNA may circulate as nucleosomes rather than cfDNA [125]. Several studies have suggested a tight correlation between DNA fragmentation patterns and nucleosome positioning with gene expression, which reflects epigenetic regulation [126,127,128]. Using the fragmentomics approach, cfDNA in cancer patients has higher fragmentation patterns and mutant sequences are enriched in shorter cfDNA fragments compared to healthy controls, which are mainly contributed by hematopoietic cells [129,130,131]. CfDNA fragmentation patterns have also provided an in vivo nucleosome footprint, which may provide clues for determining TOO [132]. The important cfDNA fragmentomics-related analytical methods are beyond the scope of this review and can be reviewed elsewhere [132]. In parallel to the technological advancements and a better understanding of cfDNA fragmentation, recent years have seen exponential interest and efforts in searching for cfDNA fragmentomics-based biomarkers, which are highly promising for applying non-invasive cfDNA-based liquid biopsy [133]. This has led to the emergence of several potential cfDNA fragmentomics-based biomarkers, including fragment sizes, preferred ends, end motifs, single-stranded jagged ends, and nucleosomal footprints [132]. Cf-DNA fragmentomics biomarkers are utilized in blood-based tests for early screening and therapeutic response monitoring of colorectal cancer patients [134], breast cancer [135], pancreatic cancer [136], and others.

#### 3.2.4. Chromatin Remodeling and Profiling

Segments of chromatin can be described as in the ‘open’ or ‘closed’ states based on compaction to modulate accessibility to DNA for replication, transcription, and repair, serving as one of the primary mechanisms behind tissue-specific gene expressions. The chromatin remodeling process is carried out by large multiprotein complexes known as chromatin remodelers. Chromatin remodelers function to modify, reposition, and restructure chromatin, impacting its compaction level and consequently influencing DNA accessibility to transcription factors, replication machinery, and repair enzymes. Chromatin remodelers utilize energy derived from ATP hydrolysis to perform their functions and are broadly classified into four leading families based on their ATPase subunits and structural characteristics. Briefly, these families are SWI/SNF (Switch/Sucrose Non-Fermentable), ISWI (Imitation Switch), CHD (Chromodomain Helicase DNA-binding), and INO80 (INOsitol requiring 80). In general, chromatin remodeling mechanisms involve sliding nucleosomes and removing or exchanging histone variants within nucleosomes. The specific functions and mechanisms of these remodelers are discussed in detail in other reviews [137].

Chromatin remodelers are linked to cancers via gene expression dysregulation and genomic instability. For example, mutations in ARID1A/B (AT-rich interactive domain-containing protein 1A/B) are among the most common aberrancy in human cancers. ARID1A is a vital component of the SWI/SNF chromatin remodeling complex, and its mutation is associated with ovarian clear cell carcinoma [138], endometrial cancer [139], and gastric cancer [140]. SMARCB1 (SWI/SNF-related, matrix-associated, actin-dependent regulator of chromatin, subfamily b, member 1) is another core component of the SWI/SNF chromatin remodeling complex that is commonly mutated. Mutation of SMARCB1 is implicated in several cancer types, such as malignant rhabdoid tumors [141,142] and other aggressive cancers that primarily affect children [143]. Mutation in the CHD1 gene, encoding Chromodomain Helicase DNA-binding Protein 1, is also reported in prostate cancer [144] and breast cancer [145]. CHD proteins influence chromatin remodeling by interacting with multiple chromatin-modifying complexes instead of forming their own subunit complexes [137]. CHD1 protein detaches DNA from the histone octamer and binds between the two DNA gyres [146]. The ATPase domain of the CHD1 protein then promotes the translocation of DNA towards the nucleosome dyad (center of nucleosomal DNA), thereby loosening the first DNA gyre and remodeling the nucleosome [146].

Although not classically thought of as a chromatin remodeler, another histone methyltransferase known as enhancer of zeste homolog 2 (EZH2) also plays an important role in chromatin remodeling and tumorigenesis. EZH2 is a component of polycomb repressive complex 2 (PRC2), which belongs to the family of Polycomb group (PcG) proteins complex that functions to maintain the spatial patterns of homeotic box (Hox) gene expression through transcriptional repression, also known as the chromatin ‘off state’ [147]. EZH2 catalyzes histone H3 on lysine 27 (H3K27) trimethylation in the nucleus mediates chromatin compaction followed by transcriptional repression of downstream genes [148]. Overexpression of EZH2 is also found in breast, lung, and prostate cancers [149,150]. Understanding the unique roles and functions of chromatin remodelers in cancer is an area of active research. It may provide valuable insights into the molecular underpinnings of tumor development and progression. Chromatin remodeling has emerged as an emerging area for biomarker development. Mutations or altered expression levels of chromatin remodelers themselves can serve as biomarkers. However, due to its complexity, chromatin remodeling-based biomarkers require high-throughput and precise analytical tools.

## 4. Overview of Technological Advances in Epigenetics cfDNA Liquid Biopsy

### 4.1. Methylation Detection Methods

Currently, common methods of epigenetics analysis of cfDNA generally utilize polymerase chain reaction (PCR)-based or combined PCR amplification and sequencing-based techniques. The methods of detection of epigenetics biomarkers described below are summarized in Figure 2 and Table 1. Currently, DNA methylation is the most common epigenetic biomarker. PCR-based methods offer versatile and sensitive approaches for analyzing DNA methylation of cfDNA. DNA undergoes bisulfite or enzymatic treatment to detect cfDNA methylation. These methods are based on converting unmethylated cytosines into uracils, which behave like thymine during PCR amplification. During PCR, uracil (originally unmethylated cytosine) pairs with adenine, and the original methylated cytosines, which remain unchanged, pair with guanine [151]. This process results in the amplification of the converted DNA, enabling further analysis. The principle of enzymatic treatment is similar to bisulfite treatment, except it utilizes a series of enzymes instead of sodium bisulfite. After bisulfite or enzymatic conversion, cfDNA can be analyzed using sequencing or methylation-specific PCR (MSP) to determine the methylation status of specific DNA regions. Sequencing after bisulfite conversion is also known as whole genome bisulfite sequencing (WGBS) [151]. The streamlined process of cfDNA extraction and bisulfite conversion into a single process for DNA methylation was developed in-house by our group and known as Methylation-On-Beads (MOB) [152]. This patented technique is well established and used extensively in our laboratory for plasma-based cfDNA methylation biomarker screening and development [152,153]. Another method is methyl-CpG-binding domain sequencing (MBD-Seq), which uses the principle that the human methyl-binding domain 2 (MBD2) protein’s high affinity and specificity for methylated CpGs on double-stranded DNA allows for efficient and robust methylation capture with minimal background noise [154,155]. This method can provide near-complete coverage of the CpG methylome as compared to array-based technologies. However, using MBD-seq for methylome-wide association studies is often fiercely criticized due to its potential limitations and misconceptions such as inefficient enrichment, poor resolution, and limited specificity of CpG methylation [155,156,157]. Another methylation detection method, which is usually confused with MBD-Seq, is methylated DNA immunoprecipitation followed by sequencing (MeDIP-seq). MeDIP-seq utilizes antibody-based affinity capture, which is not specific for CpG methylation [157]. Previous reports have shown that MeDIP-seq has lower performance and higher sequence bias than MBD-seq [158].

### 4.2. cfDNA Fragmentation Methods

In fragmentomics, a PCR-based approach is also important for analyzing cfDNA fragment sizes. There are many ways to analyze cfDNA fragments, which are reviewed in depth by Liu et al. [124]. Each method has its characteristics and advantages across different resolutions. Several PCR-based cfDNA fragments analysis methods can be used, such as quantitative PCR (qPCR), digital droplet PCR (ddPCR), multiplex PCR, and PCR-based library preparation for next-generation sequencing (NGS). Quantitative PCR can quantify specific fragment sizes of cfDNA using primer pairs targeting regions of interest. Digital droplet PCR utilizes thousands of individual droplets or wells, allowing for precise quantification of specific cfDNA fragment sizes. Multiplex PCR-based fragment size analysis amplifies multiple target regions or fragment sizes in a single assay. PCR-based library preparation methods, such as PCR amplification with barcoded primers, are used to generate sequencing libraries from cfDNA fragments for NGS analysis. Another cfDNA fragmentation analysis method is whole genome sequencing (WGS) 3-10X coverage [159,160]. Amongst the numerous methods for cfDNA fragments analysis, a significant advancement in liquid biopsy known as the DELFI (DNA evaluation of fragments for early interception) is an innovative approach for non-invasive cancer detection and monitoring [130]. DELFI leverages the characteristic fragmentation patterns of cfDNA associated with cancer to detect the presence of tumors and track disease progression [161].

### 4.3. Histone PTMs, Nucleosome Repositioning, and Chromatin Remodeling Methods

Analytical techniques of histone PTMs, nucleosome repositioning, and chromatin remodeling are not mutually exclusive. Histone PTM highly influences nucleosome repositioning and, in turn, influences chromatin states and remodeling. Many techniques are often utilized to complement one another for better characterization and to provide more information [162]. Traditionally, chromatin immunoprecipitation (ChIP) is a commonly used technique to study histone modifications and DNA–protein interactions. It involves cross-linking DNA and proteins, shearing the DNA into fragments, and then immunoprecipitation of DNA–protein complexes using antibodies specific to the histone modification of interest. The purified DNA can then be analyzed by PCR, qPCR, or sequencing (ChIP-seq) to identify the genomic regions associated with specific histone modifications. Other methods include Western blotting and mass spectrometry. Western blotting can detect specific histone modifications across a sample using antibodies specific to those modifications. However, this method must consider the genomic context in which those modifications occur. Mass spectrometry can analyze the complex mix of post-translational modifications (PTMs) on histones, providing detailed information about the type and extent of modifications. This technique is invaluable for discovering novel histone modifications and understanding their combinations and dynamics.

Although there are many methods to characterize nucleosome positioning, the most common technique stems from the concept of cutting DNA between nucleosomes and mapping protected nucleosomal DNA regions. The most frequently used method is MNase-seq. This method comprises chromatin digestion to mononucleosomes by micrococcal nuclease, isolation of nucleosomal DNA and fragments followed by deep sequencing. Unlike transcription factor or histone PTM immunoprecipitation approaches, the challenge with the analysis of nucleosome position mapping is the ability to determine the space between nucleosomes. This requires a single-base pair resolution, which requires higher sequencing coverage and is particularly important for large genomes. Understanding the mechanisms and regulation of nucleosome repositioning, especially in diseased states, is still at an early stage with much to explore compared to other epigenetic mechanisms.

There are many ways to capture the profile of chromatin remodeling. Methods that utilized identification of protein–DNA interaction complexes include the traditional CHIP-seq (chromatin immunoprecipitation) and the newer CUT&RUN (cleavage under targets and release using nuclease) and CUT&TAG (cleavage under targets and tagmentation). Another approach based on identifying open chromatin regions across the genome is ATAC-seq (assay for transposase-accessible chromatin using sequencing) and DNase-seq (DNase I hypersensitive sites sequencing). ATAC-seq and DNase-seq are enzyme-dependent methods that map functional genome elements actively involved in gene regulation. Lastly, the 3C (chromatin conformation capture) technique and its derivatives (4C, 5C, Hi-C, and others) are used to study the spatial organization of chromatin in the nucleus. This method relies on identifying regions that are physically close to each other in the 3D space of the nucleus, which can reveal looping interactions between promoters and enhancers. Another method known as ATAC-Me (assay for transposase-accessible chromatin and DNA methylation) is an integrated method to probe DNA methylation and chromatin accessibility from a single DNA fragment library to help achieve chromatin profiling [163].

## 5. Clinical Applications of cfDNA Liquid Biopsy and Epigenetics in Oncology

Before the development of liquid biopsy, invasive tissue biopsy was the gold standard for obtaining confirmatory diagnosis based on pathology and molecular studies performed on tissue samples. Originating from normal cell turnover and pathological processes, cfDNA presents a unique opportunity for monitoring and understanding various conditions, not limited to cancer. One of the pivotal roles of cfDNA lies in prenatal testing. By analyzing cfDNA in the blood of pregnant women, non-invasive prenatal tests (NIPT) can detect genetic abnormalities in the fetus, such as Down syndrome, with high accuracy and without the risks associated with invasive procedures like amniocentesis. This application has revolutionized prenatal care, allowing for early detection and intervention strategies that can significantly impact pregnancy outcomes and parental preparedness. Beyond prenatal diagnostics, cfDNA is gaining ground in transplant medicine. It can monitor organ transplant rejection by detecting donor-derived cfDNA in the recipient’s bloodstream. An increase in the levels of donor-specific cfDNA can indicate organ rejection before clinical symptoms appear, enabling timely intervention and potentially improving graft survival. Moreover, cfDNA has potential applications in detecting infectious diseases, monitoring autoimmune conditions, and understanding aging and cell senescence. Its wide-ranging utility underscores the importance of cfDNA as a tool for advancing personalized medicine, improving disease detection and monitoring, and ultimately enhancing patient care.

In recent years, liquid biopsy has emerged as a powerful and promising multi-purpose tool in oncology. In oncology, while ctDNA (a subset of cfDNA) is specifically sought for cancer-related alterations, non-tumor-derived cfDNA analysis can also provide insights into treatment-related changes, immune responses, and overall tumor burden. The integration of epigenetic analysis into circulating free DNA (cfDNA) technologies offers a promising yet underexploited avenue compared to its genetic analysis counterparts. Epigenetic markers offer a more nuanced understanding of gene regulation and expression directly reflective of tissue-level events without requiring invasive access to diseased tissues. These biomarkers, including DNA methylation, histone modifications, nucleosome positioning, cfDNA fragmentation patterns, and chromatin remodeling, manifest on the circulating DNA fragments in plasma, providing tissue-specific insights. The ability to pinpoint these unique epigenetic signatures within cfDNA, coupled with the dynamic nature of these markers, allows for a liquid biopsy approach that can trace the origin of these fragments and forecast phenotypic alterations within the local tissue environment and microenvironment. This approach may reveal critical information that is inaccessible through genetic mutations alone.

Among the various epigenetic mechanisms explored for biomarker development, DNA methylation currently stands as the predominant epigenetic biomarker utilized within cfDNA-based liquid biopsy technologies, particularly in oncology. The burgeoning field of epigenetic-based biomarkers through liquid biopsy technology spans several critical areas of medical application, including disease screening, early detection, disease monitoring and surveillance, prognostication, identification of minimal residual disease, and the targeting of therapeutic interventions. This diverse range of applications underscores the transformative potential of epigenetic analysis in refining our approach to disease management, offering a more detailed and dynamic picture of disease progression and response to treatment. Table 2 shows a list of FDA-approved and FDA breakthrough device designation DNA methylation-based biomarkers, Table 3 shows a list of non-FDA methylation-based cfDNA liquid biopsy biomarkers in development with potential for clinical use, and Table 4 shows histone modifications-based biomarkers in development with potential clinical use.

### 5.1. Early Detection and Screening

With the development of analytical advancements in liquid biopsy technology, the clinical application of liquid biopsy continues to expand and now includes early cancer detection. One of the most exciting applications of liquid biopsy currently undergoing rigorous development at its beginning stages is the multi-cancer early detection (MCED) test. MCED blood tests were first explored through a prospective interventional study known as DETECT-A (Detecting Cancers Earlier Through Elective Mutation-Based Blood Collection and Testing) using an early version of a multi-analyte blood test incorporating DNA and protein biomarkers [171]. Subsequently, several DNA methylation-based biomarkers for the MCED test were developed as listed in Table 3. Notably, the GRAIL Galleri MCED test is the first public cfDNA blood-based MCED liquid biopsy test that uses machine learning to detect and predict the origin of cancer. The Galleri GRAIL MCED test is available commercially for USD 949 and screens for a signal shared by more than 50 types of cancers [171,203,204]. It has recently revealed the results of its first prospective study (PATHFINDER), which analyzed 6621 asymptomatic adults aged 50 years or older and showed promising results supporting the feasibility of MCED screening for cancer [191]. In this study, a cancer signal was detected in 92 (1.4%) of 6621 participants; 35 (38%) of 92 were diagnosed with cancer (true positives), and 57 (62%) had no cancer diagnosis. Of those without a cancer signal detected, 6235 (95.5%) of 6529 were true negatives, 86 (1.3%) were false negatives, and 208 (3.2%) did not have a cancer-status assessment at the end of the study. This gives a positive predictive value (PPV) of 38%, negative predictive value (NPV) of 98.6%, specificity of 99.1%, and number needed to screen (NNS) of 189. Within 12 months after enrolment, 122 cancers were diagnosed in 121 participants, of which 35 (29%) had a cancer signal detected by MCED test (19 [53%] solid tumors and 17 [47%] hematological malignancies). Of these 35 participants, 26 (74%) had cancers that do not have a USPSTF screening recommendation. This study also showed that the MCED test identified 16 types of cancers, of which 12 cancers do not have USPSTF recommendations. Another major GRAIL trial, PATHFINDER 2, is underway to validate the initial promising result. This study enrolled 140,000 healthy volunteers aged 50–77 from England who had not been diagnosed with cancer in the last 3 years.

Concerning specific types of cancers, colorectal cancer has seen the most advancement in early cancer detection applications using liquid biopsy [164,174]. Most recently, Guardant Health revealed the results of its prospective, observational, multi-center study, the ECLIPSE trial, which evaluates a cfDNA blood-based screening test for colorectal cancer (SHIELD blood-based test) in average-risk individuals. This test utilizes cfDNA genomic alterations, aberrant methylation status, and fragmentomic patterns as biomarkers. The ECLIPSE study showed an 83% sensitivity for colorectal cancer and 90% specificity for advanced neoplasia. However, it is important to note that the current state of cfDNA liquid biopsy tests for colorectal cancer is not sensitive to precancerous polyps [174]. We have also demonstrated the promising potential of several DNA methylation markers, including A Disintegrin-like Metalloproteinase with Thrombospondin type 1 motif 1 (ADAMTS1), Basonuclein 1 (BNC1), leucine-rich repeat and fibronectin type III domain containing 5 (LRFN5), and Peroxidasin (PXDN) in pancreatic cancer for early detection, and ADAMTS1, BNC1, and calcium voltage-gated channel subunit alpha1 G (CACNA1G) in malignant risk stratification of intraductal papillary mucinous neoplasms (IPMNs) at our laboratory [104,180,181,205].

### 5.2. Therapeutic Targets

Currently, FDA-approved epigenetic anticancer strategies include inhibition of DNMT, HDAC, enhancer of zeste homolog 2 (EZH2), and isocitrate dehydrogenase 1 (IDH 1). The application of these epigenetic anticancer drugs is mostly limited to hematologic malignancies. In solid tumors, the therapeutic effect is variable due to the underlying epigenetic heterogeneity or lack of biomarker-driven targeted therapies. To date, there are nine Food and Drug Administration (FDA)-approved epigenetic anticancer drugs listed in Table 5. Currently, epigenetic anticancer drug treatment strategies are approached through combination therapy that targets different epigenetic mechanisms. This is due to the challenges from epigenetic monotreatment, which are attributed to resistance and limited activity. Most combination therapy clinical trials are ongoing and discussed in other reviews [206].

Azacitidine was first approved by the FDA in 2004 for treating MDS, specifically for patients with refractory anemia or refractory anemia with excess blasts [207]. Since then, the indications of epigenetic anticancer agents have expanded. Azacitidine is also used off-label to treat chronic myelomonocytic leukemia (CMML). Although the use of epigenetic agents in solid cancers is limited, many clinical trials are ongoing to explore its promising potential in solid cancers. Recently, the FDA has accepted and granted priority review for the new drug application for vorasidenib for the treatment of IDH-mutant diffuse glioma, with a Prescription Drug User Fee Act (PDUFA) action date set for 20 August 2024. Other epigenetic targets, such as bromodomain and extra-terminal motif (BET) inhibitors, are also in the early stages of development and hold promise for hematological and solid cancer treatment [208].

### 5.3. Minimal Residual Disease (MRD)

MRD testing is rapidly evolving, and new technologies and tests are continuously being developed and evaluated for clinical use. There are no well-established epigenetic-based MRD tests, but there are several in the pipeline with ongoing clinical trials and growing interest included in Table 3 [209]. Notably, in CRC, Singlera Genomics identified six DNA methylation markers designated as ColonAIQ, which showed promising results [176]. Two clinical trials utilizing ColonAIQ are underway to evaluate for postoperative recurrence monitoring of CRC (Clinical Trial ID: NCT05444491) and postoperative relapse and adjuvant chemotherapy efficacy evaluation (Clinical Trial ID: NCT05536089). Another MRD test using ctDNA methylation sequencing for multiple myeloma by Methylgene Tech is ongoing (Clinical Trial ID: NCT05578625). Colvera MRD is another laboratory-developed test (LDT) that utilizes real-time PCR tests to detect DNA methylation of BCAT1 and IKZF1 genes [210,211]. It is currently only offered as an LDT, an in vitro diagnostic test designed, manufactured, and used within a single laboratory. For the surveillance of non-muscle-invasive bladder cancer and upper tract urothelial carcinoma, the Bladder EpiCheck test is a novel urine assay that utilizes PCR-based assay to analyze the degree of DNA methylation in a group of genes [182,183,212,213]. In 2023, GRAIL, in collaboration with Astra Zeneca, entered the field of MRD by unveiling a new blood-based tumor-agnostic, methylated ctDNA MRD assay. This assay showed potential for use in six hematological malignancies: diffuse large B-cell lymphoma (DLBCL), follicular lymphoma (FL), mantle cell lymphoma (MCL), acute myeloid leukemia (AML), chronic lymphocytic leukemia (CLL), and multiple myeloma (MM). The study showed that GRAIL’s methylation technology had a cancer detection rate of 92% in patients with relapsed or refractory disease across the six hematological malignancies and a 96% accuracy in determining the hematologic malignancy subtype [192,193].

## 6. Challenges in the Translation of Epigenetics into cfDNA Liquid Biopsy

### 6.1. Test-Related Factors Affecting Sensitivity, Specificity and Accuracy

#### 6.1.1. Limitations of Pre-Analytical Methods

Although the prospects of liquid biopsy seem countless and promising, its wide usage and application are not without challenges. One of its primary challenges lies in the low concentration of cfDNA. In healthy donors, cfDNA concentration in plasma is in the range of 5–50 ng/mL, with a median value of 5 ng/mL [214,215,216]. In cancer patients, cfDNA in plasma is usually higher. Although cfDNA in plasma among cancer patients is usually below 100 ng/mL, it may vary widely and can be up to 1000 ng/mL plasma [217,218]. Depending on the stages and type of cancer, the fraction of ctDNA can exceed 10% of the total cfDNA in patients with advanced-stage cancers and as low as less than 0.1% in patients with early-stage cancers [219,220,221]. Cf-DNA has a short half-life of between 16 min and 2.5 h, although it may be longer when bound to protein complexes or inside membrane vesicles [222,223,224]. Collection methods, handling, and storage of blood or any body fluids that contain cfDNA are also crucial steps that are often overlooked and can significantly influence the quality and outcomes of cfDNA analysis by introducing contaminants [152,225]. For example, blood collected from patients should be stored in the appropriate collection tubes with adequate preserving solutions in the right temperature and conditions, followed by centrifugation into three layers of red blood cells, buffy coat, and plasma [226]. Current blood-based liquid biopsy methods generally require a high amount of blood collection and may be as much as 30–80 mL of blood [174]. All these factors should be considered when evaluating the sensitivity and specificity of the tests.

#### 6.1.2. Technical Analysis of cfDNA Liquid Biopsy

Besides pre-analytical issues, the major challenge is identifying and characterizing unique signatures that can be used as reliable biomarkers for the tissue of origin (TOO), early cancer detection, screening, monitoring, or surveillance of specific cancer types. In the pursuit of precision medicine, especially in oncology, the accurate identification and differentiation of cfDNA and ctDNA are paramount. Both cfDNA and ctDNA represent non-invasive biomarkers that circulate in the blood, offering a ‘liquid biopsy’ glimpse into the molecular makeup of tumors. However, their utility hinges on our ability to accurately distinguish between them, as they carry distinct implications. Identifying unique molecular signatures such as genetic mutations, epigenetic alterations, or unique fragment sizes can help distinguish ctDNA from the background of cfDNA. Such precise differentiation is vital, as ctDNA carries tumor-specific alterations that can inform the presence of cancer and molecular characteristics, enabling tailored therapeutic approaches and real-time monitoring of treatment response and disease progression.

Advancements in analytical methods, bioinformatics, and computational biology to enhance biomarker discovery and development are crucial in defining the impact of liquid biopsy in clinical applications. One of the most important steps in the liquid biopsy bioinformatics pipeline is the ability to eliminate the effects of contaminants or noise that may confound the true signals from biomarkers of interest and influence the accuracy and reliability of analysis. This effect can be mitigated through several strategies in the pipeline, such as bioinformatics filtering of non-specific biomarkers, normalization techniques, quality control measures using blank samples or spike-in controls, and validation studies. Although epigenetics has emerged as a promising cfDNA biomarker in precision oncology, combining epigenetics-based biomarkers with other molecular markers, such as cancer-associated proteins and genomic alterations in liquid biopsy, may improve the sensitivity and specificity of the tests.

#### 6.1.3. Handling of Liquid Biopsy Results and Data

Another significant area of concern and ambiguity involves the ethical and legal ramifications associated with using liquid biopsy technology. This includes challenges related to obtaining patient consent, safeguarding data privacy, adhering to regulatory standards, and ensuring fair access to testing. The interpretation of liquid biopsy results and determining the next course of action for ambiguous outcomes are frequently debated issues in liquid biopsy technology. The absence of a standardized protocol for handling such results can give rise to psychological concerns, particularly regarding the possibility of false positives in early detection and surveillance liquid biopsy tests.

#### 6.1.4. Accessibility and Barriers

Although liquid biopsy is an attractive technology attributed to minimal procedure-related side effects and a non-invasive and convenient approach, there are significant barriers to the accessibility and cost effectiveness of these tests. In high-income developed countries, liquid biopsy tests are deemed cost-prohibitive, not readily available, and require skilled personnel and infrastructure with high turnaround time for processing. Certain groups, especially the low socioeconomic and underserved, are affected most among the general population. For example, within the United States, access to novel technologies in medicine is not shared equally across all stakeholders due to many barriers such as payer type, discrimination of health care, mistrust of medical establishments, lack of familiarity, difficulty in terminology, and uncertainty around technology performance [227]. In low-to-middle-income nations, the challenge to implementing liquid biopsy is more substantial.

#### 6.1.5. Cost Effectiveness

In terms of cost, a systematic literature review to summarize the current health economic evidence for liquid biopsy suggested that liquid biopsy is cost-effective in selecting treatments for lung cancer patients and in screening and early detection of colorectal, gastric, breast, brain, and cancers [228]. However, it is important to interpret these findings within the context of the modeling assumptions used in each study, and further research is necessary to evaluate associated costs and health outcomes comprehensively. For example, another study evaluated the cost effectiveness of liquid biopsy for colorectal cancer screening in patients who refused traditional screening methods. Based on the model used in this study, the most cost-effective screening strategy was colonoscopy, with an incremental cost-effectiveness ratio of USD 28,071 per life-year gained. The colonoscopy–liquid biopsy hybrid had the greatest gain in life-years gained but had an incremental cost-effectiveness ratio of USD 377,538. Hence, the cost of liquid biopsy would have to be reduced by 66% (from USD 949 to USD 324) for the colonoscopy–liquid biopsy strategy to become cost effective [229].

Another example is that adding an annual MCED test would cost USD 5421.00 for patients and is predicted to provide an individual 0.34 years of quality-adjusted life. Although the MCED test is expected to increase screening costs, improved detection of cancers at early stages may potentially improve long-term health outcomes and reduce cancer treatment costs, resulting in a value-based price of USD 1196.00 at USD 100,000/quality-adjusted life-year willingness-to-pay threshold. However, the MCED test needs high specificity since false positives may lead to unnecessary further testing, adding to the cost [230].

## 7. Future Directions

### 7.1. Global Cancer Burden and Disparity

Based on the most recent data from 2022 released by the World Health Organization (WHO)’s cancer agency, the International Agency for Research on Cancer (IARC), there is a substantial growing disparity in the global cancer burden impacting underserved populations [231]. Most of the cancer burden falls on low- and middle-income countries. Despite being a preventable cancer, cervical cancer remains the primary cause of cancer-related deaths among women in 37 countries, primarily in sub-Saharan Africa. Many lower-income countries are experiencing a sharp rise in the incidence of lung, colorectal, and breast cancers in the setting of the increasing prevalence of risk factors associated with economic development, such as smoking, unhealthy diet, alcohol use, obesity, physical inactivity, and lower fertility. This is particularly striking in breast cancer according to human development. There is a higher incidence (1 in 12 women) but lower mortality (1 in 71 women) in countries with a high human development index (HDI), whereas there is a lower incidence (1 in 27 women) but higher mortality (1 in 48 women) in countries with a low HDI [231]. WHO’s global survey of health benefit packages revealed significant global disparities in cancer services [232]. Based on GLOBOCAN data from 2022, approximately 20 million cancer cases were newly diagnosed, and 9.7 million people died from the disease worldwide [233]. By 2050, the number of cancer cases is predicted to increase to 35 million based solely on projected population growth.

With the increasing global cancer burden, low- and middle-income countries (LMICs) have limited resources to respond to the overwhelming challenges that cancer brings to national health systems. There is an urgent need to address cancer disparities worldwide. CfDNA liquid biopsy holds great potential to reshape the approach towards cancer management worldwide on many levels due to its non-invasive and convenient approach. Although current applications of cfDNA-based technologies seem to benefit only a certain population with higher income or in developing nations where these technologies originate, there is a huge potential to design and develop methods of cfDNA analysis that can be tailored to what works in different countries. For example, developing point-of-care PCR-based cfDNA liquid biopsy testing for screening and early detection would be useful in middle- or low-income countries compared to sequencing-based approaches, where complex logistics such as infrastructure and expert personnel may be limited. Due to the convenience of cfDNA liquid biopsy, blood or body fluids can be collected and transported from rural areas without requiring high-level training and, hence, are more accessible and practically feasible to a larger population.

### 7.2. Integration of Artificial Intelligence and Machine Learning Workflow

**Figure 3:**
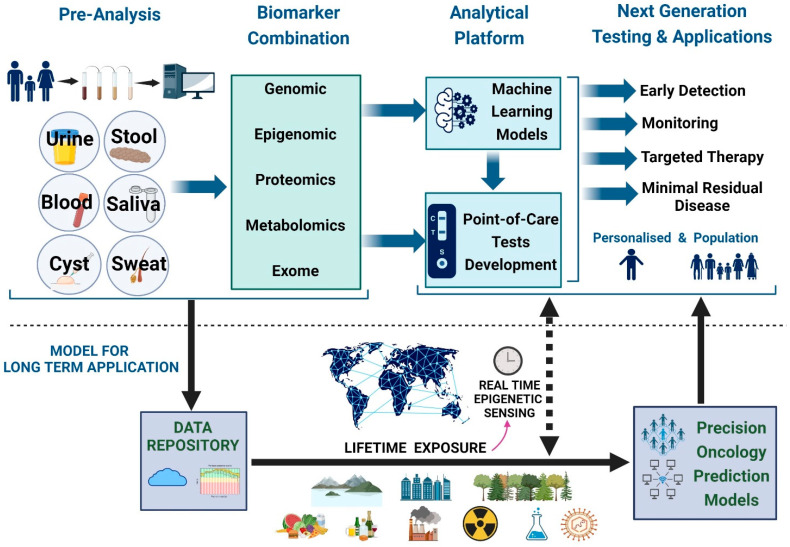
**cfDNA Liquid Biopsy Analytical Pipeline in Precision Oncology**. Molecular biomarkers can be harnessed from a patient’s bodily fluids and stool samples. These biomarkers can be combined, and when integrated with machine learning, they can be used to develop tests for early disease detection, monitoring of residual disease, and targeted therapy. The vast molecular data from liquid biopsy can be stored in a long-term repository. These data can be integrated with real-time epigenetic sensing of long-term lifetime exposures and can be used to refine precision oncology prediction models for next-generation testing and applications.

The massive amount of data obtained from liquid biopsy can be potentially harnessed to revolutionize early cancer detection and management. Using artificial intelligence, large tumor-derived omics datasets (such as genomics, epigenetics, fragmentomics, and proteomics) can be integrated and analyzed to identify complex patterns that can further provide crucial insights into cancer evolution. Different machine learning algorithms, such as network-based multi-task learning models [234], deep learning [235], and conjunctive Bayesian networks [236], have been explored for early cancer detection and minimal residual disease surveillance [237,238]. Integrative analysis of various omics data types is being increasingly applied in MCED [133,239]. For example, Cristiano and colleagues demonstrated that integrating fragmentation profiles (such as fragmentation size and coverage) with mutation detection in cfDNA enhances the sensitivity of detecting multiple cancers using low-coverage whole genome sequencing at nine times the coverage [130]. Machine learning can provide promising platforms for a wide range of clinical applications. In-depth discussion of the current application of various machine learning methods in liquid biopsy is discussed in other review articles [238]. Leveraging machine learning offers a crucial framework for integrating lifelong environmental and lifestyle exposure. This integration can enhance prediction accuracy and offer decision-making guidance, ultimately aiming to improve patient outcomes as depicted in Figure 3.

### 7.3. Personalized Medicine and Therapeutic Implications

cfDNA liquid biopsy opens an exciting new avenue to personalize the management of cancer and other diseases to specific individuals. Liquid biopsy bypasses the need for invasive procedures to obtain tissue samples, which may sometimes not be easily accessible or if samples are inadequate. Liquid biopsy may provide insights into the systemic changes occurring throughout the different organ systems rather than just the limited information the actual diseased tissue provides. Using machine learning, information from liquid biopsy may help build and train models to predict possible adverse effects from therapy based on cfDNA signatures. This would help tailor the therapeutic approach for the best possible outcome, providing more meaningful clinical trials. With a proper workflow in place for transportation and processing, liquid biopsy collection is easier and faster. This brings convenience to patients living in rural areas and may reduce the treatment delay by substituting traveling time for in-person visits and follow-ups. Implementing cfDNA liquid biopsy in large, diverse populations provides the opportunity to create a repository for long-term timeline-based molecular changes that may otherwise not be detected. These repositories can reflect unique molecular changes in specific groups or individuals based on geography, which can be further personalized to the region and person based on exposures, races, ethnicities, and social backgrounds.

## 8. Conclusions

cfDNA liquid biopsy technology offers a minimally invasive window into the dynamic systemic molecular changes accumulated throughout a lifetime. It allows us to perceive biology from a different angle and reveals knowledge and understanding of diseases from a lens that has yet to be explored. cfDNA has emerged as a promising tool in advancing precision oncology, expanding the boundaries, and transforming the conventional views of health and disease. The versatility of this technology creates an ideal platform for building a powerful network that can pave the way to reduce global cancer disparities and improve therapeutic and health outcomes in cancer care. The acceleration of cfDNA liquid biopsy advancement has sparked a simultaneous surge of interest in epigenetics in recent years. Due to its agile and dynamic nature, epigenetic changes can be captured early during cancer development years before the diagnosis. The promise and potential of epigenetics have placed it at the forefront of unraveling and deciphering complexities in cancer development. DNA methylation is the most widely utilized epigenetic biomarker among the different epigenetic features. However, others, such as histone PTMs, nucleosome repositioning, cfDNA fragmentation, and chromatin remodeling and profiling, are gaining momentum and more attention as genomic technologies evolve. The integration of machine learning in the workflow of cfDNA liquid biopsy and epigenetics has revolutionized the field of early cancer detection and screening with the development of DNA methylation-based MCED tests, which can simultaneously identify tissue of origins and screen multiple cancers. The frontiers of epigenetic applications in minimal residual disease, surveillance, monitoring, and targeted therapy will continue to blossom alongside cfDNA liquid biopsy technology. However, it is important to note that epigenetics-based cfDNA liquid biopsy development and implementation are not without challenges, and will inevitably raise skepticism as is with all other new technologies. This approach, however, will expand the boundaries of clinical applications and transform cancer management beyond its traditional paradigm.

## Figures and Tables

**Table 1:** Methods of detection of epigenetics-based cfDNA biomarkers.

Epigenetics-Based cfDNA Biomarkers	Methods of Detection
DNA Methylation	Methylation-specific quantitative polymerase chain reaction (MSQPCR) Whole Genome bisulfite sequencing (WGBS) Methyl-CpG-binding domain sequencing (MBD-Seq) Enzymatic converted sequencing (Enz-Seq) Methylated DNA immunoprecipitation followed by sequencing (MeDIP-seq)
cfDNA Fragmentation	DNA evaluation of fragments for early interception (DELFI) Droplet digital polymerase chain reaction (ddPCR) Multiplex PCR Quantitative PCR (qPCR)
Histone Post-translation Modifications (PTM)	Western blot Mass spectrometry Chromatin immunoprecipitation (CHIP)–sequencing and polymerase chain reaction (PCR)
Chromatin Remodeling	CHIP sequencing Cleavage under targets and release using nuclease (CUT&RUN) Cleavage under targets and tagmentation (CUT&TAG) Assay for transposase-accessible chromatin with high-throughput sequencing (ATAC-Seq) DNase I hypersensitive sites sequencing (DNase-Seq) Chromatin conformation capture (3C, 4C and Hi-C)
Nucleosome Positions	Micrococcal nuclease digestion with deep sequencing (MNase-Seq)

**Table 2:** FDA-approved and FDA breakthrough device designation DNA methylation-based liquid biopsy biomarkers.

FDA-Approved Methylation Markers in Liquid Biopsy					
Test (Year Granted)	Cancer Type	Biofluid Sample	Epigenetic Biomarker	Performance	Application
Cologuard (2014) [1,164]	CCR	Fecal matter	NDRG4 and BMP3 methylation (+other non-epigenetic)	SN: 92.3% (vs. FIT- 73.8%) SP: 86.6% (vs. FIT- 94.9%)	Screening/early detection
Epi proColon (2016) [165]	CCR	Blood	SEPT 9 methylation	SN: 68.2% SP: 78.8% PPV: 2.4% NPV: 99.7%	Screening/early detection
**FDA Breakthrough Device Designation Methylation Markers in Liquid Biopsy**					
**Test** **(Year Granted)**	**Cancer Type**	**Biofluid Sample**	**Epigenetic Biomarker**	**Performance**	**Application**
HelioLiver (2019) [166]	Liver (HCC)	Blood	Multiple methylation markers	SN: 85% (for any stage) and 76% (for early stages, 1 and 2) SP: 91%	Screening/early detection
IvyGene Liver (2019) [167]	Liver	Blood	ctDNA methylation markers	SN: 95% SP: 97.5%	Screening/early detection
UriFind Bladder Cancer Detection Kit (2021) [168]	Bladder and Urothelial	Urine	ONECUT2 and VIM methylation	SN: 88.1% (in patients with hematuria) and 91.2% (in patients with suspected Bladder Cancer) SP: 89.7% (hematuria) and 85.7% (suspected bladder cancer)	Screening/early detection
Bladder CARE (2023) [169]	Bladder	Urine	SIM2, N SIM2, NKX1-1, and TRNA-Cys methylation	SN: 93.5% SP: 92.6% PPV: 87.8% NPV: 96.2%	Screening/ early detection
EarlyTect Bladder Cancer (2023) [170]	Bladder	Urine	PENK methylation	SN: 84.2% SP: 95.7%	Screening/ early detection and monitoring of recurrence
Galleri (2019) [171]	Multiple	Blood	ctDNA methylation (unspecified)	SP: 99.3–99.5% SN: 51.5–63% Accuracy in locating tissue-of-origin: 88.7–93%	Screening/ early detection, locating tissue of origin
OverC Multi Cancer Detection Blood Test (2023) [172]	Multiple	Blood	ctDNA methylation markers	SN: 69.1% SP: 98.9% Accuracy in locating tissue of origin: 83.2%	Screening/ early detection

SN: sensitivity, SP: specificity, PPV: positive predictive value, NPV: negative predictive value.

**Table 3:** Non-FDA methylation liquid biopsy biomarkers in development.

GASTROINTESTINAL CANCERS					
Test	Cancer Type	Biofluid Sample	Epigenetic Biomarker	Performance	Application
Earlytect Colon Cancer [173]	CRC	Fecal matter	SDC2 methylation	SN: 90.2% (for all stages) and 89.1% (for early stages 0–2) SP: 90.2%	Screening/early detection
SHIELD by Guardant Health [174]	CRC	Blood	Aberrant methylation status and fragmentomic patterns	SN: 83% (for CRC) SN: 13% (for advanced precancerous polyp) SP: 90% (for CRC and advanced precancerous polyp)	Screening/early detection
Colovera [12,175]	CRC	Fecal matter	BCAT and IKZF1 methylation	SN for recurrence: 75% (for stage 2 cancer) and 70.6% (for stage 3 cancer) The odds ratio of recurrence with a positive test: is 14.4 (vs. 6.9 with CEA).	Monitoring minimal residual disease and recurrence
ColonAIQ [176]	CRC	Blood	6 DNA methylation markers	SN: 86% SP: 92%	Monitoring minimal residual disease and recurrence
MYO1-G methylation [177]	CRC	Blood	MYO1-G methylation	SN: 84.3% SP: 94.5%	Screening/early detection and disease monitoring
HCCBloodTest [178]	Liver (HCC)	Blood	SEPT9 methylation	SN: 76.7% SP: 64.1%	Screening/early detection
epiLiver [179]	Liver	Blood	VASH2, CHFR, GRID2IP, CCNJ, F12 methylation	SN: 84.5% SP: 95%	Screening/early detection
(Unnamed) Combination panel for ADAMTS1, BNC1, LRFN5, and PXDN [104,180]	Pancreatic	Blood	ADAMTS1, BNC1, LRFN5, and PXDN methylation	All 4 genes: SN: 100% SP: 90% ADAMTS1 & BNC1: SN: 97.3% SP: 91.6%	Screening/early detection
(Unnamed) Combination panel for ADAMTS1, BNC1 and CACNA1G [181]	Intraductal papillary mucinous neoplasms (IPMNs)	Blood	ADAMTS1, BNC1, and CACNA1G methylation	All 3 genes: SN: 83.0% SP: 80.0% BNC1 & CACNA1G: SN: 71.0% SP: 97.0% ADAMTS1/BNC1: SN: 77.0% SP: 83.0%	Malignant risk stratification of precancerous lesion
**GENITOURINARY CANCER**					
Bladder EpiCheck [182,183]	Non-Muscle invasive bladder cancer	Urine	Multiple methylation markers	SN: 68.2% (overall) and 91.7% (excluding low-grade non-invasive papillary carcinoma) SP: 88% (overall) NPV: 99.3% (excluding low-grade non-invasive papillary carcinoma)	Surveillance/monitoring minimal residual disease and recurrence
	Upper tract urothelial carcinoma (UTUC)	Urine	Multiple methylation markers	SN: 83% SP: 77% NPV: 59% PPV: 61%	Surveillance/monitoring minimal residual disease and recurrence
AssureMDx [184]	Bladder	Urine	TWIST1, ONECUT2, and OTX1 methylation (+other non-epigenetic biomarkers)	SN: 97% SP: 83% NPV: 99.6–99.9%	Screening/early detection
**LUNG CANCER**					
Epi proLung BL Reflex Assay [185]	Lung	Blood	SHOX2 and PTER4 Methylation	SN: 78% SP: 96%	Screening/early detection
PulmoSeek [186]	Lung	Blood	ctDNA methylation	SN: 100% (6–20 mm nodules), 97.1% (Stage 1 malignancy), 100% (solid nodules), 94.7% (part-solid nodules), 96.4% (ground-glass nodules)	Screening/early detection
**MULTI-CANCER DETECTION**					
Cancer Radar [4,187,188]	Multiple	Blood	ctDNA methylation (unspecified genes)	SP: 97.9% SN:80.7% (all-stage cancer) and 74.5% (early-stage cancer); accuracy in locating the tissue-of-origin: 89.1% (all-stage cancer) and 85.0% (early-stage cancer)	Screening/early detection, locating tissue of origin
PanSeer [189]	Multiple (colorectal, lung, liver, stomach, esophagus)	Blood	ctDNA methylation (unspecified genes)	SN: 88% SP: 96%	Screening/early detection
IvyGeneCORE [190]	Multiple (breast, colon, liver, lung)	Blood	ctDNA methylation (unspecified genes)	SN: 89–95% SP: 96–100%	Screening/early detection
GRAIL Galleri [191]	Multiple	Blood	ctDNA methylation (unspecified genes)	SP: 99.1% PPV: 38% NPV: 98.6% NNS: 189 Accuracy of Cancer Signal Origin: -First correct: 85% -First of second correct: 97%	Screening/early detection, locating tissue of origin
GRAIL in collaboration with Astra Zeneca [192,193]	Six types of hematologic malignancies	Blood	ctDNA methylation (unspecified genes)	SN: 92% Accuracy in determining the hematologic malignancy subtype: 96%	Surveillance/monitoring minimal residual disease and recurrence

SN: sensitivity, SP: specificity, PPV: positive predictive value, NPV: negative predictive value, NNS: number needed to screen.

**Table 4:** Histone modifications-based biomarkers with potential for clinical translation.

Histone Modifications	Cancer Types	Potential Clinical Outcome
Recurrent mutation of H3K27M [194,195,196]	Pediatric glioblastoma	Worse prognosis
Lower levels of H3K9ac, H3K9me3, H4K16ac and H4K20me3 [197]	Non-small cell lung cancer	Poor prognosis (recurrence and distant metastasis)
Loss of H4K20me3 [198]	NSCLS (squamous cell carcinoma and adenocarcinoma)	Poor prognosis in adenocarcinoma
Lower levels of H3K4me2 and H3K18ac [199]	Prostate cancer	Poor prognosis
Lower levels of H3K9ac, H3K18ac, H4K12ac, H3K4me2, H4K20me3, and H4R3me2 [200]	Breast cancer	Poor prognosis
Lower levels of H3K4me2, H3K9me2 and H3K18ac [201]	Pancreatic adenocarcinoma	Poor prognosis
Higher levels of H3K9me3 [202]	Gastric adenocarcinoma	Poor prognosis (tumor stage, lymphovascular invasion, cancer recurrence, and poor survival rate)
Upregulation of EZH2 causing trimethylation of histone H3 at lysine 27 (H3K27me3) [148]	Metastatic prostate and breast cancer	Potential therapeutic target

**Table 5:** Food and Drug Administration (FDA)-approved epigenetic anticancer drugs.

Target and Mechanisms	Drugs	FDA Approval Indications
**DNMT inhibitor:** Inhibits DNA methylation, leading to hypomethylation	Azacitidine	Myelodysplastic syndrome Acute myeloid leukemia - Myelodysplasia-related changes - Adult patients who achieved complete remission (CR) or complete remission with incomplete blood count recovery (CRi) following intensive induction chemotherapy and who are not able to complete intensive curative therapy - Patients 65 years or older or who have comorbidities that preclude the use of intensive induction chemotherapy (in combination with venetoclax)
	Decitabine	Myelodysplastic syndrome
**HDAC inhibitor:** Inhibits histone deacetylase, affecting chromatin structure and gene expression, which increases the acetylation of histone proteins, leading to an open chromatin structure	Belinostat	Relapsed or refractory peripheral T-cell lymphoma (PTCL)
	Panobinostat	Multiple myeloma who have received at least two prior regimens, including bortezomib and an immunomodulatory agent (in combination with bortezomib and dexamethasone).
	Vorinostat	Cutaneous T-cell lymphoma (CTCL) Refractory or relapsed peripheral T-cell lymphoma (PTCL)
	Romidepsin	Cutaneous T-cell lymphoma (CTCL) Refractory or relapsed peripheral T-cell lymphoma (PTCL)
**EZH2 inhibitor** (histone methyltransferase enzyme): Inhibits the addition of methyl groups to histone H3 lysine 27 (H3K27), leading to gene silencing and chromatin compaction	Tazemetostat	Epithelioid sarcoma—16 years and older with metastatic or locally advanced disease. Relapsed or refractory follicular lymphoma (FL)
**IDH1 inhibitor:** Inhibition leads to the production of oncometabolite 2-hydroxyglutarate (2-HG) instead of α-KG in the citric acid cycle; 2-HG inhibits histone demethylases and TET (ten-eleven translocation) enzymes involved in DNA demethylation, affecting histone methylation and DNA methylation	Ivosidenib	Relapsed or refractory acute myeloid leukemia (AML) with a susceptible IDH1 mutation
	Enasitenib	Relapsed or refractory acute myeloid leukemia (AML) with an isocitrate dehydrogenase-2 (IDH2) mutation
